# Faltering lemming cycles reduce productivity and population size of a migratory Arctic goose species

**DOI:** 10.1111/1365-2656.12060

**Published:** 2013-02-19

**Authors:** Bart A Nolet, Silke Bauer, Nicole Feige, Yakov I Kokorev, Igor Yu Popov, Barwolt S Ebbinge

**Affiliations:** 1 Department of Animal Ecology, Netherlands Institute of Ecology (NIOO-KNAW)PO Box 50, Wageningen, NL-6700, AB, The Netherlands; 2 Department of Bird Migration, Swiss Ornithological InstituteSempach, 6204, Switzerland; 3 Laboratory of Biological Monitoring, Extreme North Agricultural Research Institute (RAAS)Komsomolaskaya Street 1, Norilsk, 663302, Russia; 4 Laboratory of Biogeocenology and Historical Ecology, A.N. Severtsov Institute of Ecology and Evolution (RAS)33 Leninskij prospekt, Moscow, 119071, Russia; 5 Team Animal Ecology, Alterra Wageningen-URPO Box 47, Wageningen, NL-6700, AA, The Netherlands

**Keywords:** bird migration, climate change, dark-bellied brent goose, density dependence, reproductive success

## Abstract

**1.** The huge changes in population sizes of Arctic-nesting geese offer a great opportunity to study population limitation in migratory animals. In geese, population limitation seems to have shifted from wintering to summering grounds. There, in the Arctic, climate is rapidly changing, and this may impact reproductive performance, and perhaps population size of geese, both directly (e.g. by changes in snow melt) or indirectly (e.g. by changes in trophic interactions).

**2.** Dark-bellied brent geese (*Branta bernicla bernicla* L.) increased 20-fold since the 1950s. Its reproduction fluctuates strongly in concert with the 3-year lemming cycle. An earlier analysis, covering the growth period until 1988, did not find evidence for density dependence, but thereafter the population levelled off and even decreased. The question is whether this is caused by changes in lemming cycles, population density or other factors like carry-over effects.

**3.** Breeding success was derived from proportions of juveniles. We used an information-theoretical approach to investigate which environmental factors best explained the variation in breeding success over nearly 50 years (1960–2008). We subsequently combined GLM predictions of breeding success with published survival estimates to project the population trajectory since 1991 (year of maximum population size). In this way, we separated the effects of lemming abundance and population density on population development.

**4.** Breeding success was mainly dependent on lemming abundance, the onset of spring at the breeding grounds, and the population size of brent goose. No evidence was found for carry-over effects (i.e. effects of conditions at main spring staging site). Negative density dependence was operating at a population size above c. 200 000 individuals, but the levelling off of the population could be explained by faltering lemming cycles alone.

**5.** Lemmings have long been known to affect population productivity of Arctic-nesting migratory birds and, more recently, possibly population dynamics of resident bird species, but this is the first evidence for effects of lemming abundance on population size of a migratory bird species. Why lemming cycles are faltering in the last two decades is unclear, but this may be associated with changes in winter climate at Taimyr Peninsula (Siberia).

## Introduction

Unlike resident species, vital rates and hence the equilibrium population sizes of migratory species may be affected by conditions encountered on the breeding, wintering, and stopover sites (Newton 2004). For resident and migratory species alike, a change in population size may in itself affect survival and reproduction through negative density dependence (Newton 1998). However, as population limitation can occur in more than one part of the world, achieving an understanding of density dependence is difficult in migratory species (Newton 2004). Moreover, conditions experienced in one part of the world can potentially affect the performance of the migrants in other parts (carry-over effects; Marra, Hobson & Holmes 1998; Norris & Taylor 2006; Harrison *et al*. 2011). A multi-factorial approach is therefore needed to unravel the causes of variation in vital rates of migratory species.

Swan and goose species (Anserini), many of which breed in northern regions and winter in the temperate zone, seem to be well suited for such a multi-factorial approach to the study of vital rates and equilibrium population sizes of migrants. Because young of the year spend the winter with their parents (Cramp & Simmons 1977), both reproductive and annual mortality rate can be quantified on the wintering grounds. Most swan and goose species have increased over the past 50 years, which has been attributed to a higher winter survival because of shooting restrictions (Ebbinge 1991) as well as improved feeding conditions related to agricultural changes (Jefferies 2000; Fox *et al*. 2005; Van Eerden *et al*. 2005) on the wintering grounds. Concurrently, reproductive rates have dropped in some of these species, suggesting a density-dependent response in breeding output (Madsen, Cracknell & Fox 1999). Density dependence determines to what numbers these populations will ultimately rise, and is therefore also of management interest, because conflicts with agriculture on the wintering grounds have greatly intensified (van Eerden 1990).

Voles and lemmings have long been known to exhibit multi-annual population cycles, especially in more northerly regions (Elton 1942). Lemmings (*Lemmus* spp. and *Dicrostonyx* spp.) generally show population cycles with peaks every 3–5 years (Kokorev & Kuksov 2002; Gilg, Hanski & Sittler 2003; Krebs 2010). They play an important role in the Arctic food web, and are the preferred prey of generalist predators like Arctic foxes (*Vulpes lagopus* L.), skuas and gulls (Lari) (Gauthier *et al*. 2004). Predation pressure by these generalist predators on alternative prey like Arctic-nesting birds varies with lemming abundance, and the birds' reproductive output varies accordingly (Summers 1986). During the last 25 years or so, vole and lemming cycles are fading out in some northerly regions, which has been attributed to changes in winter conditions (Bierman *et al*. 2006; Ims, Henden & Killengreen 2008; Kausrud *et al*. 2008; Gilg, Sittler & Hanski 2009). Through increased predation pressure on alternative prey, such collapses of lemming cycles have been suggested to have negative consequences for resident breeding birds (Kausrud *et al*. 2008). Most birds in areas where lemmings occur are, however, migratory (Gilg & Yoccoz 2010), and hence the question arises whether such negative consequences also exist for migratory birds.

**Fig. 1:**
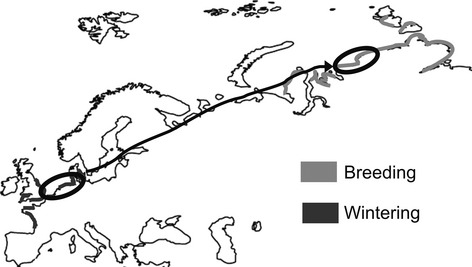
Distribution of dark-bellied brent geese during winter, migration and breeding. Ovals indicate spring departure site in the Wadden Sea and the artic tundra in Western Taimyr, with the Pyasina river delta (with Mys Vostochny) located in the centre (after Madsen, Cracknell & Fox 1999).

**Fig. 2:**
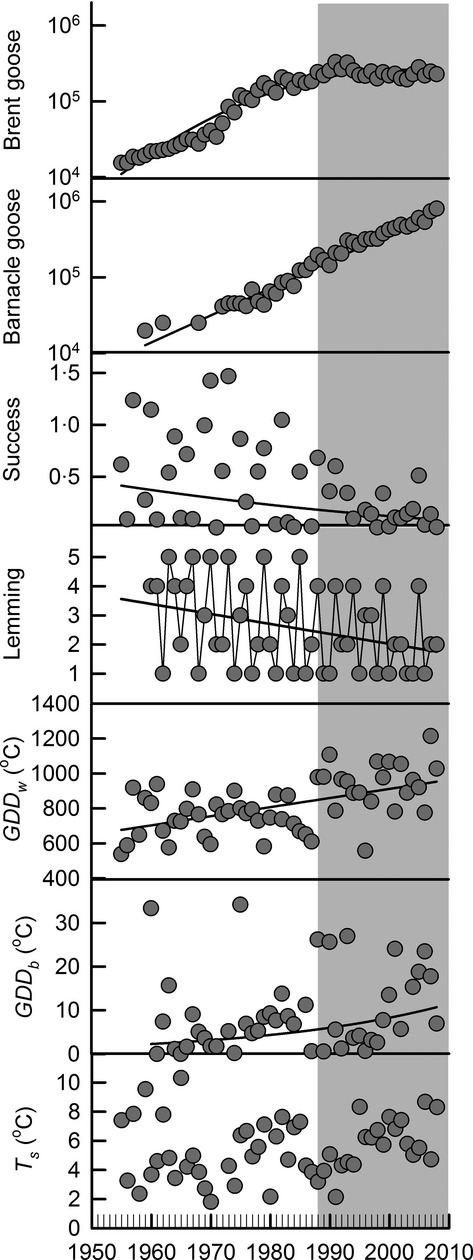
Population development of dark-bellied brent goose and Russian/Baltic barnacle goose between 1955 and 2008. Lines indicate significant trends (see Results). Over this time period, brent goose breeding success as well as lemming abundance in Taimyr decreased, whereas spring temperature sums (expressed as growing degree days), both at the spring staging site in the Wadden Sea (*GDD*_*w*_) and at the Taimyr breeding grounds (*GDD*_*b*_), increased. Average temperature during the gosling phase in summer (*T*_*s*_) did not change. The shaded area indicates the period since 1988 when overall lemming abundance and climate changed most markedly.

Dark-bellied brent geese (*Branta bernicla bernicla* L.; hereafter: brent geese) breed on coastal arctic tundra and winter in the temperate zone of western Europe (Fig. 1). This population has undergone a 20-fold increase since the 1950s (Fig. 2, top panel). Unlike many other goose species, the population decreased after the mid-1990s, suggesting overcompensating density dependence (Ebbinge *et al*. 2002). However, its reproduction strongly fluctuates in concert with the approximately 3-year lemming cycle (Blomqvist *et al*. 2002; Ebbinge & Spaans 2002; Summers 1986). When lemmings are scarce, reproductive success is invariably low, and therefore a density-dependent relationship of the number of first-winter brent geese was only borne out after selecting only those years with presumed low predation at the breeding grounds (Ebbinge *et al*. 2002). In contrast, an earlier analysis of the same population, only covering the growth phase of the population, concluded that there was no evidence for density dependence (Summers & Underhill 1991). Instead, these authors suggested that population growth could be predicted from a constant survival rate and a reproductive rate, oscillating with lemming abundance.

However, even in years of high lemming abundance, breeding success of brent geese may be low (e.g. 1961, 1967, 1994), so for a full understanding other factors like weather conditions should be considered as well (Ebbinge 1989). A late spring in the arctic reduces Brent goose reproduction (Barry 1962; Lindberg, Sedinger & Flint 1997; Spaans *et al*. 1998), and in summer, the gosling phase is a critical period in the survival of juveniles (Morrissette *et al*. 2010).

To complicate matters further, pre-breeding conditions, and particularly those at the spring departure site may have carry-over effects on the breeding output of migratory birds (Ebbinge & Spaans 1995; Schmutz, Hobson & Morse 2006; Spaans *et al*. 2007). In the last few decades, these conditions have changed quite considerably, especially on the main spring departure site in the Wadden Sea (Fig. 1; van der Graaf *et al*. 2006). Moreover, the Russian/Baltic barnacle geese (*Branta leucopsis* Bechstein), that make use of the same departure site in spring, have increased exponentially since 1960 (Fig. 2). These birds have also prolonged their spring staging period: whereas they used to leave at the end of March, many now stay until early May (Eichhorn *et al*. 2009). Competition with barnacle geese may reduce the conditions for spring fuelling of brent geese, because brent geese leave later, and are less inclined to graze on previously grazed patches than barnacle geese (Engelmoer *et al*. 2001; Stahl *et al*. 2006).

Hence, apart from population density, changes in summer and winter season in the Arctic, as well as changes in winter and spring conditions outside the Arctic may all impact the performance of these arctic-nesting migrants. Here we apply a longer time series than Summers & Underhill (1991), including a phase of decline, to test whether their conclusion still holds that the population trajectory of dark-bellied brent geese can be understood from a reproductive rate that is solely dependent on lemming abundance, without invoking density dependence. Their hypothesis would predict that the population decline is due to faltering lemming cycles.

## Materials and methods

### Study population

Dark-bellied brent geese breed mainly on the arctic tundra coast of Taimyr peninsula (Siberia), and winter 5000 km to the southwest along the western European coast. In spring, virtually the whole population gathers in the Wadden Sea for pre-migratory fuelling (Fig. 1).

Population numbers were derived from the mid-winter (i.e. January) counts conducted under the auspices of the Goose Specialist Group of Wetlands International. In winter, this population confined to coastal areas in the Netherlands (20%), France (40%) and England (40%), with much smaller numbers in Denmark and Germany, and no major change in its winter distribution has occurred while the population size increased (Madsen, Cracknell & Fox 1999). Its concentrated occurrence enables full coverage of census counts, with accuracy likely to be within 10% of the true total (Fox *et al*. 2010). This is substantiated by the finding that annual survival estimated from these counts is within 1% of that estimated from re-sightings of individually marked brent geese using a Jolly-Seber approach (Ebbinge 1992; Ebbinge *et al*. 2002).

There is some evidence for local shifts in population distribution in spring with changes in population size (Ebbinge 1992; Engelmoer *et al*. 2001). Such information from the breeding grounds is lacking, but given the loose colony structure and nomadic nature of brent geese in summer (Ebbinge 2004), we expect breeding density and hence intraspecific competition to increase with population size.

### From breeding success to population development

We used an information-theoretical approach to determine the main environmental factors affecting population productivity (Morrissette *et al*. 2010). Then we used a general linear model containing the best explanatory factors to predict breeding success in a given year. We subsequently combined these with published estimates of survival to project population development under a few selected ecological scenarios in order to separate the effects of changes in lemming cycles and population density.

### Survival and breeding success

Proportions of juveniles were assessed by ageing birds at all major wintering sites, with sampling effort (25–50% of the population) increasing with the increase in population. We used these proportions of juveniles, weighted for population size per country, to subdivide the census population in different age classes, and to calculate population breeding success. Because brent geese are capable of breeding at 2 years of age (Ebbinge *et al*. 2002), we subdivided the census *N*_*c*_ in a given year *t* in three age classes, namely first-winter birds *N*_*f*_, second-winter birds *N*_*s*_, and older birds *N*_*a*_. The number of first-winter birds was calculated from the proportion of first-winter birds *p*_*t*_ as *N*_*f,t*_ = *p*_*t*_. *N*_*c,t*_. The number of second-winter birds was derived from the number of first-winter birds in the previous year as *N*_*s,t*_ = *s*_*t*_. *N*_*f,t-1*_, where *s* is survival of birds > 0·5 year old (survival after the first 6 months was considered constant; Ebbinge *et al*. 2002). Survival was modelled to be lower in the first decades of the study as hunting was still allowed in Denmark until 1972 (Ebbinge 1985). Crude survival analyses yielded estimates of *s* of 0·81 before 1972, and 0·85 after 1972 (Summers & Underhill 1991). Considering the whole period (both before and after 1972), *s* was, however, estimated to be higher (0·86) (Ebbinge *et al*. 2002; Summers & Underhill 1991), so we used *s* of 0·81 and 0·86 for before and after 1972 respectively. Subtracting the first- and second-winter birds from the census number gave the number of older birds *N*_*a,t*_:


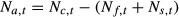
eqn 1

Breeding success *b*_*t*_ was subsequently calculated as:


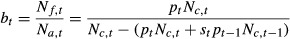
eqn 2

*b*_*t*_ was square-root transformed to normalize the data.

### Lemming abundance

In brent geese, reproduction strongly fluctuates in concert with the lemming cycle (Blomqvist *et al*. 2002; Ebbinge & Spaans 2002; Summers 1986), Relative lemming abundance (*L*) was derived from numbers of lemmings caught in snap-traps ordered along transects in different tundra habitats in summer (June-August). The main species is Siberian lemming (*Lemmus sibiricus* Kerr), with collared lemming (*Dicrostonyx torquatus* Pallas) being about an order of magnitude less abundant. *L* was classified into five categories according to the total number of lemmings caught per 100 trap-days (1: < 1; 2: 1–3; 3: 4–10; 4: 11–30; 5: > 30) (Kokorev & Kuksov 2002). We used the lemming index for arctic tundra in Western Taimyr (zone of c. 100 km along the coast of the Kara Sea between 80 and 90˚E) (Chernov 1985) as provided for 1960–2001 by Kokorev & Kuksov (2002). We complemented this series for 2002–2008 from snap-trap data collected at Mys Vostochny (74˚08′ N, 86˚44′ E) in the Pyasina delta, also in the arctic tundra of Western Taimyr (1200–1500 trap-days per summer) (Rykhlikova & Popov 2000). Due to its logarithmic nature, the lemming index is robust to slight changes in methodology: the lemming index determined at Mys Vostochny in 1993–1995 was identical to that given by Kokorev & Kuksov (2002). No trapping data were available for 2003, but observations (http://www.arcticbirds.net) suggest a lemming index of 1, in accordance with the notion that the previous year, 2002, had been a lemming peak year (that did not grow to its full potential; Tulp 2007).

### Climatic effects

Pre-breeding conditions, and particularly those at the spring departure site may have carry-over effects on the breeding output of brent geese (Ebbinge & Spaans 1995; Spaans *et al*. 2007). In another Arctic-nesting goose species, body stores were correlated with the growing degree days (*GDD*) around spring departure (Duriez *et al*. 2009). As a proxy for departure conditions from the pre-migratory fuelling site we therefore took growing degree days (*GDD*_*w*_; subscript refers to wintering grounds). *GDD*_*w*_ was calculated as the sum of average daily temperature above 0 °C measured at Leeuwarden weather station (53˚12′N, 05˚47′ E) until 23 May, the mean date of mass departure of brent geese from the Wadden Sea.

Brent goose reproduction is dependent on spring conditions (Barry 1962; Lindberg, Sedinger & Flint 1997; Spaans *et al*. 1998), and because plant development is closely related to a spring temperature sum (Wang 1960), we used growing degree days in Taimyr (*GDD*_*b*_; subscript refers to breeding grounds) as a proxy for arrival conditions in the breeding grounds. *GDD*_*b*_ was calculated as the sum of average daily temperature above 0 °C measured at Dikson weather station (73˚31′ N, 80˚20′ E) until 19 June, the mean date at which 50% of the nesting brent geese are present in Taimyr (Spaans *et al*. 2007). This measure was a good proxy for the date of snow melt: *GDD*_*b*_ was negatively correlated with the date at which 50% of the snow at our transect in Mys Vostochny had melted (on average 20 June; Pearson's *R* = −0·75, *N* = 9, *P*_1t_ < 0·01).

Mean temperature during the gosling phase is correlated with the annual productivity of another Arctic-nesting goose species (Morrissette *et al*. 2010). As a proxy for the conditions during the first month after hatching, we took the average daily temperature (*T*_*s*_; subscript refers to summer) measured at Dikson weather station between 21 July (mean hatching date, Spaans *et al*. 2007) and 20 August, when all surviving goslings have fledged.

### Interspecific competition

The Wadden Sea spring staging site is also used by barnacle geese during their preparation for migration to the Baltic Sea and the Barentsz Sea (Russia) (Eichhorn *et al*. 2009), and competition with barnacle geese may reduce the conditions for spring fuelling of brent geese (Engelmoer *et al*. 2001; Stahl *et al*. 2006). The numbers of barnacle geese *M* were also derived from mid-winter (i.e. January) counts (Ganter *et al*. 1999; Van der Jeugd *et al*. 2009). Missing data in the early period with little population growth (10 years up to 1972) were linearly interpolated.

### Data analysis

For the period 1960–2008, we ran a general linear model with square-root transformed breeding success (√*b*_*t*_) as the dependent variable, lemming abundance (*L*) as a categorical predictor, and as continuous predictors the natural log-transformed brent and barnacle goose population sizes of the preceding winter ln(*N*_*t-1*_) and ln(*M*_*t–1*_), respectively, the growing degree days in the Wadden Sea and in Taimyr (*GDD*_*w*_ and log(*GDD*_*b*_) respectively), and the average summer temperature (*T*_*s*_). Because ln(*N*_*t-1*_) and ln(*M*_*t–1*_) were highly correlated (Pearson's *R* = 0·865, *N* = 49, *P* < 0·001), we did not consider these together to avoid multicollinearity. Weather data were not complete for 1971 and 1972, so we used averages over 1960–2008 (log(*GDD*_*b*_) = 0·764 for 1972, and *T*_*s*_ = 5·4 °C for 1971 and 1972). All possible 48 models were ranked according to Akaike's Information Criterion, corrected for small sample size (AICc; Burnham & Anderson 2002), using the glmulti-package in R (Calcagno & de Mazancourt 2010). Akaike weights were calculated by summing over all models; models withΔAICc > 2 were considered to have less than substantial empirical support (Burnham & Anderson 2002).

### Projected population development

We used predictive modelling to separate the effects of ecological drivers, through their effects on breeding success, on population development. We used published estimates of annual survival *s* (see Survival and breeding success) and our modelled breeding success *β*_*t*_ to derive the expected number of birds 
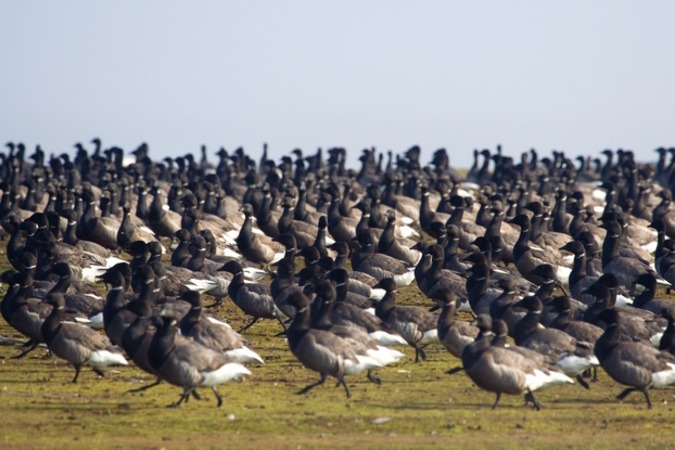
:


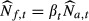
eqn 3

where


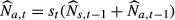
eqn 4

As (see Survival and breeding success):


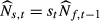
eqn 5

this gives an expected census number:


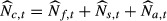
eqn 6

Such projections suffer from error propagation, and therefore we did not model the whole time series. Because we were specifically interested in the factors causing the population to level off and even decline, we used the census with maximum numbers (1991) as the start for these projections.

## Results

Since 1955, population size of the dark-bellied brent goose increased (ln(*N*_*t*_), Pearson's *R* = 0·97, *N* = 55, *P* < 0·001), but clearly levelled off and even decreased in the 1990s (Fig. 2; year^2^ was significant in polynomial regression: *t*_52_ = −9·41, *P* < 0·001). Concurrently with the increasing population size, brent goose' breeding success dropped (√*b*_*t*_, Pearson's *R* = −0·29, *N* = 54, *P* < 0·05). The lemming abundance also showed a decrease, because peaks occurred less regularly, and were less pronounced (Fig. 2; Spearman *ρ* = −0·32, *N* = 49, *P* < 0·05). In contrast to the brent goose, the Russian/Baltic barnacle goose population continued to grow in an exponential fashion (Fig. 2; (ln(*M*_*t*_), Pearson's *R* = 0·98, *N* = 40, *P* < 0·001). Spring temperature sums (i.e. growing degree days) at the breeding grounds in Taimyr increased (log(*GDD*_*b*_), Pearson's *R* = 0·34, *N* = 48, *P* < 0·02), but average temperatures during the gosling phase did not change (*T*_*s*_; Pearson's *R* = 0·17, *N* = 52, *P* = 0·23). Temperature sums at the spring departure site in the Wadden Sea also increased (*GDD*_*w*_; Pearson's *R* = 0·538, *N* = 54, *P* < 0·001). The observed changes in lemming abundance and climate were all most prominent since 1988 (shaded area in Fig. 2).

**Fig. 3:**
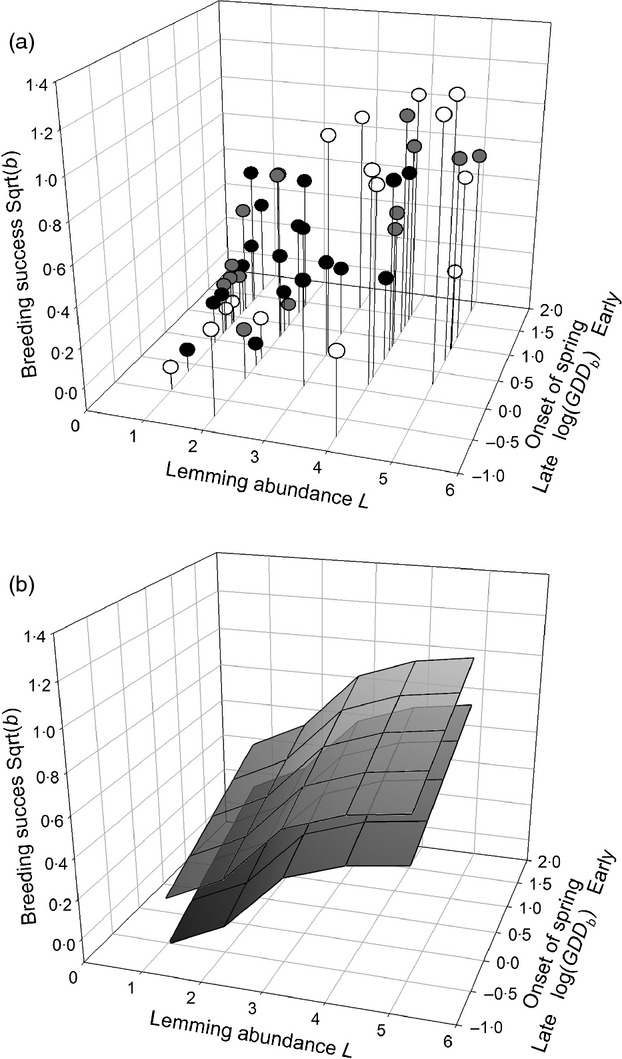
Breeding success (√*b*) as a function of lemming abundance (*L*) and spring conditions expressed as growing degree days (^o^C) at the breeding grounds in Taimyr (log*GDD*_*b*_) (a relatively low *GDD* represents a late spring). Breeding success increases with lemming abundance, and is higher when spring in Taimyr is early and, in general, when population size is low. (a) Observed values shaded by the brent goose population size ln(*N*_*t-1*_), increasing from white (< 100 000) to black (> 200 000). (b) Predicted values according to most parsimonious model (model 1 in Table 1) for population size of 15 000 (upper mesh) and 300 000 (lower mesh).

The most parsimonious model explained breeding success (√*b*_*t*_) of brent geese with lemming abundance (*L*), brent goose population size, ln(*N*_*t-1*_), and the growing degree days in Taimyr (*GDD*_*b*_). This model clearly performed better (ΔAICc > 2) than all other models (Table 1). Considering all 48 models, these variables were also best explaining the observed variation in breeding success (Table 2). The size of the brent goose population itself featured much more prominently than that of its competitor, the barnacle goose, with a sum of Akaike's weights of 0·16 for ln(*M*_*t-1*_) compared to 0·61 for ln(*N*_*t*-1_). (Note that *w*_*i*_'s of ln(*N*_*t-1*_) and ln(*M*_*t*-1_) were slightly underestimated because these variables only featured in 16 compared to 24 models for the other independent variables; ignoring multicollinearity and hence considering 24 models for ln(*N*_*t-1*_) and ln(*M*_*t*-1_) too, their *w*_*i*_ rose to 0·66 and 0·27 respectively). Weights of evidence for effects of summer temperature at the breeding grounds and growing degree days at the spring staging site were also considerably lower (Table 2). Another way to test whether there was a density-dependent effect on breeding success is to perform a type three likelihood ratio test (Sokal & Rohlf 1995) for the best performing model. This revealed that the increment in log-likelihood by adding brent goose population size ln(*N*_*t-1*_) to a model containing lemming abundance (*L*) and the growing degree days in Taimyr (*GDD*_*b*_),was marginally significant (χ_1_ = 3·82, *P* ≍ 0·05). So, breeding success of the brent geese was higher when lemmings were abundant, whereas it was lower in late springs (as indicated by a low *GGD*), and tended to be lower at large brent goose population sizes (Fig. 3).

**Table 1:** Top 5 of explanatory models of breeding success (√*b*_*t*_) of dark-bellied brent geese according to Akaike's information criterion corrected for small sample size (AICc; *n* = 49). Each model is defined by the listed independent variables: lemming abundance *L*, spring conditions at the breeding grounds in Taimyr (expressed in growing degree days, *GDD*_*b*_), spring conditions at the departure site in the Wadden Sea (*GDD*_*w*_), average summer temperature in Taimyr (*T*_*s*_), and population size of competitor species (barnacle goose, ln(*M*_*t-1*_), Models with a difference of AICc less than two are indicated in bold; *K* is the number of parameters, ℒ(*m*_*i*_|*x*) is likelihood of model *i* given the data, and *w*_i_ is its Akaike's weight. RMSD is the root mean squared deviation of observed on predicted values (of √*b*_*t*_), and *R*^2^ the proportion of the variation in observed values explained by the predicted values (of √*b*_*t*_)

Model	*K*	Scaled deviance	ΔAICc	ℒ(*m*_*i*_|*x*)	*w*_*i*_	Evidence ratio	RMSD	*R*^2^
(1) *L* – ln(*N*_*t-1*_) + log(*GDD*_*b*_)	**8**	**1·17**	**0·00**	**1·00**	**0·35**	**1·0**	**0·22**	**0·63**
(2) *L* – ln(*N*_*t-1*_) + log(*GDD*_*b*_) – *T*_*s*_	9	1·20	2·33	0·31	0·11	3·2	0·21	0·64
(3) *L* – ln(*N*_*t-1*_) + log(*GDD*_*b*_) – *GDD*_*w*_	9	1·20	2·61	0·27	0·10	3·7	0·22	0·63
(4) *L* + log(*GDD*_*b*_) – ln(*M*_*t-1*_)	8	1·17	2·64	0·27	0·09	3·8	0·22	0·62
(5) *L* + log(*GDD*_*b*_)	7	1·14	2·67	0·26	0·09	3·8	0·23	0·60

**Table 2:** Weight of evidence for effect of the six test variables on reproductive output of dark-bellied brent geese (√*b*_*t*_). Standardized coefficients of the full model give an indication of their relative effect size. Akaike weights are summed over all 48 models, and give an indication of how much support for an effect is provided by the data. The Pearson's correlation coefficient indicates the simple correlation with √*b*_*t*_

Variable	Pearson's *R*	Standardized coefficients	*w*_*i*_
Lemming class (*L*)*	0·72	0·58	1·00
Growing degree days Taimyr, log(*GDD*_*b*_)	0·26	0·29	0·94
Brent goose population size, ln(*N*_*t-1*_)	−0·35	−0·34	0·61
Summer temperature (*T*_*s*_)	−0·24	−0·13	0·24
Growing degree days Wadden Sea (*GDD*_*w*_)	−0·29	−0·11	0·24
Barnacle goose population size, ln(*M*_*t-1*_)	−0·31	0·21	0·16

* these estimates were obtained by reclassifying lemming class as 0 for *L* ≤ 2 and 1 for *L* ≥ 3.

**Fig. 4:**
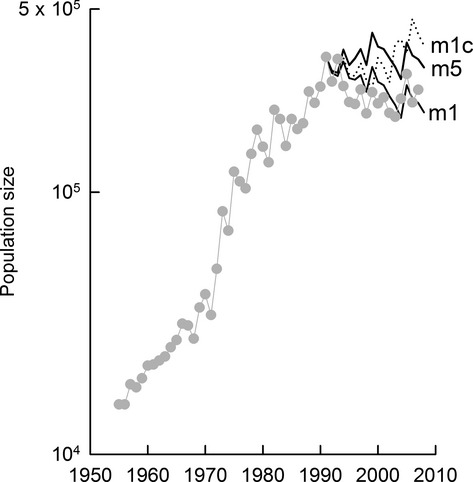
Population development of dark-bellied brent goose according to the census (dots), and projected from 1991 (when the population size was maximal) onwards (black lines). Projections are based on crude survival estimates (see Methods) and breeding success predicted from lemming abundance and spring temperature sums at the breeding grounds (m5, model 5 in Table 1), or, additionally, the brent goose population size (m1, model 1 in Table 1). m1c = model 1 but with lemming cycles continuing as before 1988. For parameter estimates see supplementary Table S1.

We used this most parsimonious model (model 1 in Table 1; for parameter estimations, see Table S1) to predict breeding success (from the environmental variables *L*, log(*GDD*_*b*_) and ln(*N*_*t-1*_)), and to project population development. The model nicely captured the decrease in the brent goose population after 1991 (Fig. 4, m1). Interestingly, the model without ln(*N*_*t-1*_), i.e. excluding negative density dependence on breeding output (model 5 in Table 1), predicted a levelling off, but not a decrease, in population size (Fig. 4). We investigated to what extent the decrease was due to the observed changes in lemming abundance *L*, which changed quite abruptly in 1988 (Fig. 2). Hence, we substituted the values for the 20 years including and following 1988 with those of the 20 years preceding 1988 in model 1. If lemming abundance had not changed after 1988, brent goose population size would eventually have increased further (Fig. 4, m1c; note that this population increase was projected even though negative density dependence on breeding output was accounted for).

## Discussion

We have identified lemming abundance, the onset of spring at the breeding grounds and population density to be the best explaining factors of population productivity in dark-bellied brent geese over the last half-century. There was little evidence for carry-over effects arising from conditions at the main spring staging site.

### Faltering lemming cycles

The levelling off and even decrease in population size of brent geese is due to a series of years with low breeding output, which is mainly the result of faltering lemming cycles during the last two decades. Although these geese do not always produce many offspring when lemmings are abundant, the converse holds: in lemming trough years following a lemming peak year, when the generalist predators are numerous and are relying on alternative prey like brent geese eggs, breeding success is consistently low (Ebbinge & Spaans 2002; Gauthier *et al*. 2004). Our projective modelling suggests that if lemming abundance had not changed, brent geese population size would eventually have increased further, despite density dependence operating.

Changes in winter climate have been suggested to cause the lemming cycles to falter (Ims, Henden & Killengreen 2008; Kausrud *et al*. 2008; Gilg, Sittler & Hanski 2009). Lemmings can reproduce under snow cover, which provides protection from predators and thermal insulation, while food plants are still accessible (Stenseth & Ims 1993; Millar 2001; Korslund & Steen 2006; Reid *et al*. 2012). Snow conditions are therefore a crucial determinant of lemming peaks (Ims, Yoccoz & Killengreen 2011). There are indications that these are changing in Taimyr. The snow cover period tends to be shorter, but snow depth has increased considerably in 1966–2007 (Bulygina, Razuvaev & Korshunova 2009), and hence these changes are not all negative for lemmings. In some recent years (2002 and 2008), lemming peaks did not seem to reach full potential, despite lemming nests being about as abundant under the snow (in 2008) as in a year that featured a lemming peak (2005) (Feige *et al*. 2012). It seems therefore particularly relevant to investigate whether rain-on-snow or melt-refreeze events occur earlier or more often (Ye, Yang & Robinson 2008; Rennert *et al*. 2009), as these events may mean that more water is perforating through the snow, potentially jeopardizing the insulation of the lemmings, and perhaps ice layers on the tundra surface render feeding grounds inaccessible (Korslund & Steen 2006; de Raad, Mazurov & Ebbinge 2011). Such icing is also thought to be responsible for the lengthening of the lemming cycle from 5–8 years at Wrangel Island (Menyushina *et al*. 2012).

### Coping with late springs

In the Arctic, the timing of snow melt, and hence the length of the summer season, varies considerably among years. Arctic-nesting geese start nesting at a somewhat later date in years when snow melts late (Prop & De Vries 1993; Cooke, Rockwell & Lank 1995; Madsen *et al*. 2006), but, relative to snow melt, commence nesting early in late springs (Barry 1962; Lindberg, Sedinger & Flint 1997; Bêty, Gauthier & Giroux 2003). Also, like in other bird species (Murphy 1986; Perrins & McCleery 1989), clutch size in geese is generally smaller in late springs (Barry 1962; Raveling 1978; but see Lindberg, Sedinger & Flint 1997; Madsen *et al*. 2006). Geese probably start nesting early relative to snow melt in late springs to enhance the survival of the goslings (Prop & De Vries 1993).

In their decision when to commence nesting, the birds face another trade-off, namely that between current and future reproductive success (Daan, Dijkstra & Tinbergen 1990). In many geese and duck species, only females incubate the eggs, and the body weight at the end of the incubation is supposedly near the critical boundary (Drent *et al*. 2003). Laying a smaller clutch may compensate for the lower pre-laying condition of the female, leading to equal body weights at the start of incubation irrespective of the onset of spring (Sénéchal, Bêty & Gilchrist 2011; Spaans *et al*. 2007). Such compensation may be only partial, as suggested by the finding that female black brants (*Branta b. nigricans* Lawrence) weighed less during incubation in a later spring (Eichholz & Sedinger 1999).

Commencing nesting at a higher snow cover in late springs would mean that poorer feeding opportunities may extend well into the incubation phase (Prop & De Vries 1993). Then female geese would have to compensate for poorer feeding conditions in the early incubation phase by spending more time feeding (black brant: Eichholz & Sedinger 1999; dark-bellied brent geese: B.A. Nolet, R.A. Bom, J. de Fouw, P.P. de Vries, B.S. Ebbinge, unpublished). Limiting the loss in body condition would clearly enhance the female's survival prospects and future reproductive success. Because leaving the nest by the female is risky for dark-bellied brent geese because of egg predation (Spaans *et al*. 2007), egg predation is expected to be heavy in late spring. So, egg survival seems to be traded-off against both gosling and female survival to make the best of a bad job.

All in all, reproduction is severely reduced in late springs. Such late springs are predicted to become less frequent (Tulp & Schekkerman 2008), and this may partly compensate the effects of faltering lemming cycles (see above).

### Density dependence

In an earlier study, Summers & Underhill (1991) did not find evidence for density dependence operating during the growth phase of the dark-bellied brent goose population (1955–1988). At the end of that period population size was just above 200 000 individuals. Our analysis, which covers another 20 years in which the population roughly fluctuated between 200 000 and 300 000 individuals, shows that there was a negative, albeit weak, effect of population size on breeding output. This suggests that density dependence only starts to have an effect at a population size above 200 000 individuals.

Recent analyses suggest density dependence is acting in some Arctic-nesting goose species like the Svalbard barnacle goose (Trinder, Hassell & Votier 2009), but not in others like the greater snow goose (*Anser caerulescens atlanticus* Kennard) (Morrissette *et al*. 2010). During the growth phase of the dark-bellied brent goose population, an increasing proportion of the birds staged at a less preferred site, suggesting that the preferred site was filled to capacity (Ebbinge 1992). If, as a consequence, a larger proportion of females leaves with a relatively low condition, this might affect subsequent population productivity (Ebbinge & Spaans 1995). However, in our analysis we did not find evidence for such carry-over effects (see Carry-over effects), and we think that density dependence, noticeable on the level of the entire population, acts mainly on the summer grounds.

The main nesting habitat of brent geese are coastal islands, that are usually also frequented by Taimyr gulls (*Larus fuscus taimyrensis* Buturlin). Only in lemming peak years, considerable numbers also nest on the mainland around nests of snowy owls (*Bubo scandiacus* L.) that only breed in such years (Underhill *et al*. 1993; Summers *et al*. 1994). There are indications that breeding distribution is despotic in both nesting habitats, with clutch sizes being larger close to gulls' nests (at least in predation years; J. de Fouw *et al*., unpublished) and close to snowy owls' nests (van Kleef *et al*. 2007). The negative density dependence may arise from these best nesting territories being filled to capacity (Rodenhouse, Sherry & Holmes 1997).

### Carry-Over and interaction effects

In a classic study, it was shown that dark-bellied brent geese that are heavy upon departure from the Wadden Sea spring staging area have a higher probability to breed successfully (Ebbinge & Spaans 1995). Contrary to the notion of ‘Arctic amplification’ (Solomon *et al*. 2007), the climatic changes on this spring staging site in the temperate region were even more pronounced than those at the Arctic breeding grounds. However, measured across the whole population we did not find an effect of spring weather conditions in the Wadden Sea on reproductive output. We also did not find evidence for a depressing effect of the increase in the competitor species, the barnacle goose. Competition between barnacle and brent geese is intense on the departure site in the Wadden Sea, with brent geese suffering more from barnacle geese than the other way around (Stahl *et al*. 2006). However, these species are spatially segregated at the breeding grounds (Cramp & Simmons 1977). Brent goose and barnacle goose population development were partly running parallel, and hence the effect of barnacle geese may have been masked by even stronger effects of brent geese population density itself.

To avoid possible spurious effects we had to reduce the number of models under consideration, and we did not investigate possible interaction effects. For instance, the effect of a late spring at the breeding grounds might be less detrimental if it coincides with a late spring at the departure site. In general, a late spring at the departure site may lead to a later departure (Duriez *et al*. 2009; Stirnemann *et al*. 2012), reducing the costs of waiting at the breeding grounds. Such effects may be small in dark-bellied brent geese, however, because departure is probably more triggered by wind conditions (Green 2001).

## Conclusion

Lemmings have long been known to affect, indirectly, population productivity of Arctic-nesting migratory birds (Summers 1986). More recently, population dynamics of resident bird species were shown to change in concert with changes in lemming dynamics, but this might be due to direct effects of snow hardness on both lemmings and resident grouse species (Kausrud *et al*. 2008). Our analysis points to faltering lemming cycles, perhaps caused by changes in the Arctic winter, as the main factor causing changes in both population productivity and population size of a migratory bird. Although in the last decade our knowledge about the dynamics of these rodents has improved, different factors seem to play a role at different places (Gilg, Hanski & Sittler 2003; Kausrud *et al*. 2008; Krebs 2010; Menyushina *et al*. 2012). Hence, much more research is needed before longer term predictions on lemming abundance and all its dependencies, sometimes apparently stretching 5000 km, can be made.

## Supporting Information

**Table S1.** Parameter estimates of two general linear models explaining reproductive output of dark-bellied brent geese (√*b_t_*) used to project brent goose population size. Model number according to Akaike's information criterion (Table 1).
