# Supplementary material for: Faltering lemming cycles reduce productivity and population size of a migratory Arctic goose species
Source: J Anim Ecol. 2013 Feb 19;82(4):804–13. doi: 10.1111/1365-2656.12060 (PMC3744762; doi:10.1111/1365-2656.12060)
Supplement: Supplementary file 1 [file jane0082-0804-SD1.docx]

**Supporting Information**

**Table S1.** Parameter estimates of two general linear models explaining reproductive output of dark-bellied brent geese (√*b_t_*) used to project brent goose population size. Model number according to Akaike’s information criterion (Table 1).

|  | Model 1 | | | Model 5 | | |
| --- | --- | --- | --- | --- | --- | --- |
| Variable | | Estimate | Standard  error | | Estimate | Standard  error |
| Intercept | | 1.294 | 0.445 | | 0.412 | 0.056 |
| Lemming class (*L*) 1 | | -0.332 | 0.056 | | -0.357 | 0.057 |
| Lemming class (*L*) 2 | | -0.193 | 0.064 | | -0.227 | 0.064 |
| Lemming class (*L*) 3 | | 0.094 | 0.082 | | 0.084 | 0.086 |
| Lemming class (*L*) 4 | | 0.195 | 0.061 | | 0.205 | 0.063 |
| Lemming class (*L*) 5 | | 0.236 |  | | 0.295 |  |
| Growing degree days Taimyr, log(*GDD_b_*) | | 0.179 | 0.057 | | 0.138 | 0.056 |
| Brent goose population size, ln(*N_t-1_*) | | -0.079 | 0.040 | |  |  |
